# Five Cases of Metastatic Gas Gangrene

**Published:** 1919-01

**Authors:** 


***Five Cases of Metastatic Gas Gangrene. Three Followed by Recovery.*** By J. V. J. Hartley. *Journal of the Royal Army Medical Corps.* August 1918.

That micro-organisms, in cases of gas gangrene, gain entrance to the bloodstream and produce lesions in distant parts of the body, is perhaps of more frequent occurrence than is generally supposed.

Case 1. Comminuted humerus with very foul and septic wound. December 6, operation. December 9, the arm below the wound became cold and areas of discoloration and bullae appeared on the forearm and hand. Arm amputated. The same evening, crepitation in the left buttock. Died December 10. At autopsy, the gluteal muscles were found to be greyish, soft and crepitant. A bacteriological examination demonstrated the presence of Bacillus aerogenes capsulatus in large numbers.

Case 2. Gun shot wound of the abdomen with fecal fistula. Further examination revealed swelling and crepitation over the right deltoid. Patient rapidly grew worse and died.

Case 3. Gun shot wound of the right arm. Arm amputated. Later pain was complained of in the right buttock and on examination there was found to be some swelling and a suspicion of deep crepitation. An incision was made to the right of the sacrum. Patient recovered.

Case 4. Admitted November 23d, with very serious condition of trench feet. The left leg was amputated midway between ankle and knee; the right leg was amputated a hand's breadth above the ankle. December 12th, slight improvement, stumps satisfactory. December 12th, still complaining of pain in buttock. Incisions were made downwards and outwards from the posterior superior spine. December 16th, buttock wounds douched daily with hydrogen peroxide and eusol gauze applied. January, 2, patient transferred to England,

Case 5. Wounded in the right buttock, right arm and right shoulder. Deep crepitation could be made out in the opposite buttock. An incision was made downwards and outwards from the left posterior superior spine of the ilium and gas and a thin bloodstained fluid escaped. General condition much improved. Recovery.

In all cases in which a bacteriological examination was made the causal organism of the metastatic infection was the B. aerogenes capsulatus. Four of the secondary lesions occurred in the buttocks and it would seem that continuous pressure due to the patient's posture was an important factor in the localizing of the metastatic infection.

In the fatal cases, 1 and 2, the intravenous injection of eusol was given early. In case 3 the extensive gas gangrene in the right buttock and thigh occurred five days after amputation of the right arm. In case 4 the metastatic infection in both buttocks developed two weeks after the amputation of the gangrenous feet. Here the operation for the secondary lesion was limited and no attempt was made to extirpate the infected muscles. One can only suggest that in these two cases the intravenous injection of eusol delayed metastasis and that, prior to secondary lesions, the patient had time to acquire immunity sufficiently powerful to overcome the disease.

